# Efficacy of repetitive transcranial magnetic stimulation combined with peripheral magnetic stimulation on movement symptom and exploration of the optimal population in Parkinson’s disease: A randomized controlled trial

**DOI:** 10.1097/MD.0000000000040689

**Published:** 2024-11-29

**Authors:** Peili Sun, Junrui Li, Haiqing Shen, Yongcheng Jiang, Xinjue Wang, Tian Xu, Lihua Shen, Xiaosu Gu

**Affiliations:** a Department of Neurology, Affiliated Hospital and Medical School of Nantong University, Nantong, China; b Department of Neurology, The People^’^s Hospital of Rugao, Nantong, China; c Xuzhou Medical University, Xuzhou, China; d Research Center of Clinical Medicine, Affiliated Hospital of Nantong University, Nantong, China.

**Keywords:** movement symptom in Parkinson’s disease, Parkinson’s disease, repetitive peripheral magnetic stimulation, repetitive transcranial magnetic stimulation

## Abstract

**Background:**

This study explores the efficacy of repetitive transcranial magnetic stimulation (rTMS) and rTMS combined with repetitive peripheral magnetic stimulation (rPMS) (hereinafter referred to as rTMS + rPMS) on motor symptoms and quality of life in Parkinson’s disease (PD), and explores whether there are differences between the two treatment methods; At the same time, analyze data from different subgroups to explore the influencing factors, in order to find the most suitable treatment group.

**Methods:**

Eighty patients with PD were randomly divided into rTMS and rTMS + rPMS groups and administered 10 Hz rTMS, and 10 Hz rTMS + 25 Hz rPMS, respectively, for 10 days. Before and after treatment, the PD Motor Function Rating Scale (UPDRS Part III, 10m Walk Timing Test, Stand Up Walk Test Evaluation Scale (TUG)) and PD Quality of Life Questionnaire (PDQ-39) were used to evaluate the motor symptoms and quality of life. After quantifying the treatment effect, a comparative analysis of the efficacy before and after treatment was conducted. Simultaneously, we divided the two treatment groups into different subgroups, compared the subgroups under the same treatment method, analyzed the relevant factors affecting the treatment method, and found the most suitable treatment group.

**Results:**

(1) After rTMS or rTMS + rPMS, all scoring scales improved compared to those before treatment (*P* < .05). Compared to rTMS, rTMS + rPMS resulted in greater improvements in overall motor function (UPDRS III) and quality of life (PDQ-39) (*P* < .05). (2) Patients with rigidity-based type as the main type may be the most suitable for these two treatment methods (*P* < .05).(3) There was no significant difference in treatment efficiency between the two treatment methods for patients with PD at different disease stages, sexes, or treatment ages(*P* > .05).

**Conclusion:**

Both rTMS and rTMS + rPMS can improve movement symptoms and quality of life in patients with PD. rTMS + rPMS was more beneficial for improving the overall motor function. Patients with rigidity-based type as the main type may be the most suitable for these two treatment methods. The therapies work in all age groups, all gender and irrespective of the disease stage with varying levodopa equivalent daily doses as well.

## 1. Introduction

Parkinson’s disease (PD) is a neurodegenerative disease common in middle-aged and older adults. The common symptoms include motor (bradykinesia, tremor, rigidity, abnormal posture, and gait) and non-motor (depression, mental symptom, and cognitive impairment) symptoms.^[1,2]^ The current main treatment method is dopaminergic drugs, such as levodopa, as a replacement therapy.^[3]^ However, drug treatments have limitations and may lead to motor complications including end-of-dose phenomena and symptoms.^[4]^ Therefore, it is important to study treatment methods other than drugs, such as repetitive transcranial magnetic stimulation (rTMS), deep brain stimulation, and transcranial direct current stimulation.

rTMS is a noninvasive neuromodulation technology that delivers repetitive magnetic pulses to specific brain areas in a short period through a stimulation coil placed on the scalp.^[5]^ rTMS regulates cerebral cortex excitability and improves motor function in PD patients. This effect is evident, and the treatment is economical and safe.^[6]^

Recent studies have suggested that the combination of rTMS and repetitive peripheral magnetic stimulation (rPMS) may have stronger and more lasting effects on corticospinal and intracortical excitability than single magnetic stimulation.^[7–9]^ Relevant studies on stroke and certain therapeutic effects have been conducted.^[10–12]^The combination of rTMS and rPMS has not yet been applied in the treatment of PD, and it is unclear whether this treatment plan can improve the therapeutic effect of PD.

However, rTMS combined with rPMS has not yet been used to treat PD, and whether this treatment regimen can improve the therapeutic effect in these patients remains unclear. Therefore, the present study investigated the therapeutic effects of rTMS and rTMS + rPMS on motor symptoms and quality of life in patients with PD, and explored whether there were differences between the two treatment methods. By comparing the therapeutic effects in patients with PD with different clinical characteristics, the factors affecting the efficacy of the two treatment methods were explored, and the most suitable population for treatment was explored.

## 2. Materials and methods

### 2.1. Research participants

**Figure 1.**
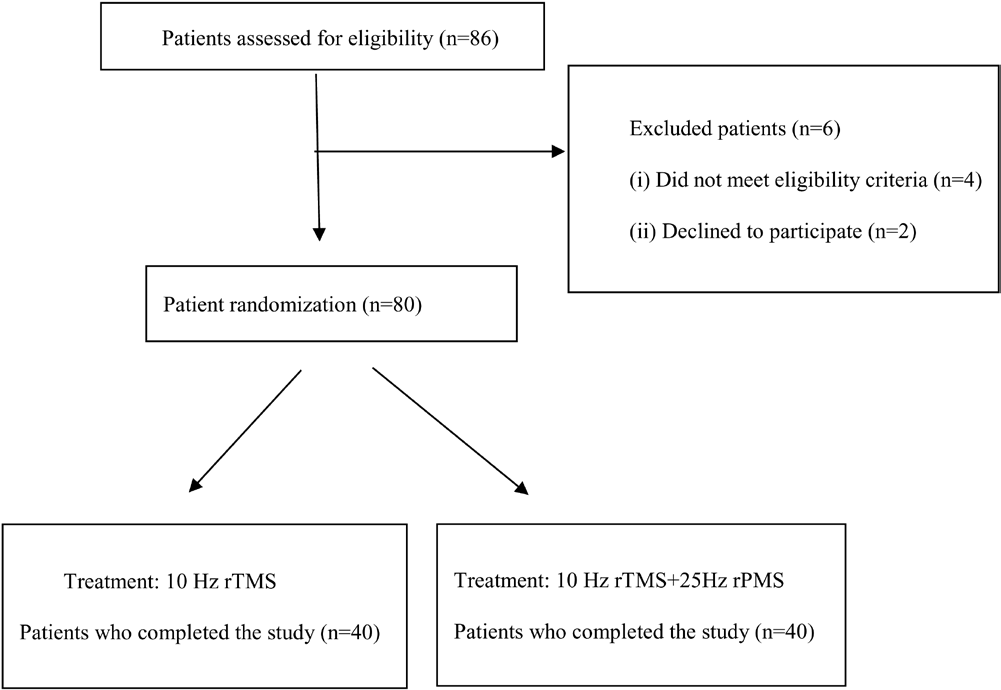
Flowchart of patient recruitment and treatment assignments.

Eighty-six PD patients who met the inclusion criteria were admitted to the Affiliated Hospital of Nantong University between September 2022 and December 2023. Among these, 6 patients were excluded because of non-research factors, leaving 80 enrolled patients (Fig. 1).

This study included patients diagnosed with PD by a PD specialist based on the 2015 International Movement Disorder Association Clinical Diagnostic Criteria for PD,^[13]^ without severe physical disease, without hearing or vision impairment, with an education level of primary school or above, who had no communication barriers, could cooperate with the treatment, could walk 10 m independently, were aware of the study, had good compliance, voluntarily participated, signed the informed consent form, and adhered to the entire treatment course.

The exclusion criteria were patients with secondary Parkinson’s syndrome (cerebrovascular disease, traumatic brain injury, intracranial space-occupying lesions, hydrocephalus, or other neurological diseases leading to Parkinson’s syndrome); Parkinson’s plus syndrome, history of craniotomy and epileptic seizures, contraindications for TMS including cardiac pacemakers or intracranial stents, history of deep brain stimulation surgery, severe cognitive impairment who could not cooperate with treatment, inability; to voluntarily participate, and lack of informed consent were excluded.

This study was approved by the Ethics Committee of the Nantong University Hospital (2022-K132-01). Written informed consent was obtained from all participants. All procedures were performed in compliance with the relevant laws and institutional guidelines.

### 2.2. Study design

This randomized, open-controlled trial was conducted by clinicians who did not participate in the efficacy evaluation. Participants were randomly divided into rTMS, rTMS and rPMS combination treatment groups in a 1:1 ratio by drawing lots and registering.

Owing to the different clinical characteristics of PD patients, which may have an impact on treatment, we further divided the 2 groups of patients into different subgroups based on their clinical characteristics: (1) Classification by disease stage: According to the revised Hoehn and Yahr (H–Y) classification, patients were classified by disease stage (early stage: H–Y grade 1–2.5; middle-late stage: H–Y grade 3–5). The higher the grade, the more severe are the symptoms. (2) Divided by sex: male and female. (3) Divided by treatment age: ≤60 and >60 years. (4) According to the type of motor symptoms, it can be divided into 3 groups: tremor-dominant (TD), akinetic-rigid dominant (PIGD), and indeterminate.^[14]^ PD type was determined according to the average tremor score/average non-tremor score ratio in Parts II and III of the Unified PD Rating Scale (UPDRS). The tremor score was assessed using items 16 (tremor), 20 (resting tremor), and 21 (tremor of hand movements or postures) of the UPDRS. Non-tremor scores were assessed using the UPDRS items 13 (fall), 14 (freezing), 15 (walking), 29 (gait), and 30 (postural stability), including the number of individual subitems in each body region. The indeterminate type was evaluated using the average UPDRS tremor score (8 items) and the UPDRS average non-tremor score (5 items). Patients with TD, PIGD, and indeterminate types were defined as those with ratios of ≥1.5, ≤1, >1.0, and <1.5, respectively. Moreover, patients with a positive numerator mean and zero denominator mean were classified as having TD, those with a numerator of zero and a positive denominator mean were classified as having PIGD, and those with both zero numerator and denominator were classified as indeterminate. Indeterminate types were excluded from analysis.

### 2.3. Research methods

#### 2.3.1. Setting stimulus plans

##### 2.3.1.1. rTMS treatment plan

The stimulation target was the primary motor cortex area (M1), and the center of the stimulation coil was vertically placed above the motor cortex area to measure the motor evoked potential and calibrate the target in real time. Once determined, precise labeling was performed on the treatment cap. Measure the resting motor threshold (RMT) to determine treatment intensity throughout the entire trial process. RMT is defined as the intensity of motor evoked potential amplitudes exceeding 50V induced by at least 5 of 10 stimuli in the first dorsal interosseous muscle.

When using rTMS, the patient is in a supine position, naturally relaxed, and the “8” shaped coil is placed in the M1 area on the healthy side of the patient, with the handle pointing towards the back or outside. The frequency was 10 Hz, the stimulation intensity was 120% RMT level, the stimulation time was 1.5 seconds, with an interval of 10 seconds, and the total stimulation pulse was 1560 pulses, lasting for 20 minutes.

##### 2.3.1.2. rPMS treatment plan

When using rPMS, the patient maintains a supine position, naturally relaxes, and the “8” shaped coil is aligned with the affected fibular muscle. A magnetic stimulation coil was placed in the abdominal muscle, and the treatment intensity was appropriate to cause significant muscle contraction. The stimulation frequency was 25 Hz and it lasted for 20 minutes.

#### 2.3.2. Magnetic stimulation therapy

The treatment for the same patient was selected at a fixed time every day, with a total of 10 magnetic stimulation treatments performed once a day, 5 times a week, for 2 weeks. Throughout the entire study period, the patient’s Parkinson’s medication and stimulation therapy settings remained unchanged.

rTMS group: Based on the conventional drug treatment, rTMS stimulation therapy was performed for 20 minutes.

rTMS + rPMS group: After 20 minutes of rPMS treatment, adjust the central stimulation site for 20 minutes of rTMS treatment. The combination of the 2 lasted 40 minutes in total.

### 2.4. Efficacy evaluation

Before treatment was started and after 2 weeks of treatment, 2 experienced neurologists unaware of the grouping evaluated the treatment efficacy in the 2 patient groups. All assessments were conducted at the same time of the day, during the “on” period after taking the medication.

The Unified PD Rating Scale-III (UPDRS-III),^[15]^ the up-and-go test scale (TUG),^[16]^ and the 10 m timed walk test (10MW) were used to evaluate the severity of movement symptoms in patients with PD. The UPDRS-III includes 14 items: speech, facial expression, tremors, rigidity, posture, and gait. Each item is scored 0–4 points, with a maximum total score of 56. The lower the score, the better is the motor function of the patient. The TUG is used to quickly assess balance and walking. In this test, the participants stood up from the chair, walked 3 m, turned around, walked back to the chair, and sat down. The 10MW assesses walking ability by measuring walking speed in meters per second. In this test, the participant started from the 0 m line and walked 10 m. The time required to travel from 2 m to 8 m was recorded. The total walking time was then divided by 6 m and measured in m/s. The average of 3 measurements was calculated.

In this study, PDQ-39 was used to evaluate the treatment status of patients with PD. The questionnaire consisted of 39 items. Each question has 5 answers and is scored from 0 to 4 points. The higher the score, the worse the patient’s quality of life.

The differences before and after treatment were calculated, and the changes in the UPDRS-III, TUG, 10MW, and PDQ-39 scores were compared and analyzed. The UPDRS-III was the main evaluation index.

### 2.5. Statistical analysis

Data were analyzed using IBM SPSS Statistics for Windows, version 26.0. Data that did not conform to a normal distribution are represented as medians (quartiles) and compared using the Wilcoxon rank-sum test. Independent samples and paired rank-sum tests were used for comparisons between and within the groups, respectively. Data that fit the normal distribution were expressed as mean ± standard deviation and analyzed using the *t* test. Count data are expressed as percentages and compared using the chi-square test. Statistical significance was set at *P* < .05.

## 3. Results

### 3.1. Comparisons of general clinical data between the rTMS and rTMS + rPMS groups

Age, disease duration, sex distribution, Mini-Mental State Examination (MMSE) score, Hamilton Depression Rating Scale score, levodopa equivalent daily dose, and H–Y classification did not differ significantly between treatment groups (*P* > .05) (Table 1).

**Table 1:** Comparison of general clinical data.

		rTMS(n = 40)	rTMS + rPMS(n = 40)	*P*
Age (years)		63.8 ± 9.87	62.62 ± 8.23	.652
Disease duration (years)		4.65 ± 2.68	4.21 ± 2.19	.529
Sex	Male	18 (45%)	25 (62.1%)	.238
	Female	22 (55%)	15 (37.9%)	
HAM-D		15.2 ± 6	16.7 ± 3.9	.383
MMSE		24.3 ± 1.2	24.06 ± 1.62	.732
LEDD (mg/d)		516 (387–668.75)	516 (350–616)	.372
H–Y classification	Early stage	16 (53.33%)	18 (62.07%)	.591
	Middle-late stage	14 (46.67%)	11 (37.93%)	

Notes: H–Y classification, the revised Hoehn and Yahr (H–Y) classification.

HAM-D = Hamilton Rating Scale for Depression; LEDD = levodopa equivalent daily dose; MMSE = mini-mental State Examination; rPMS = repetitive peripheral magnetic stimulation; rTMS = repetitive transcranial magnetic stimulation.

There were no statistically significant difference in the scores on the evaluation scales (UPDRS III, TUG, 10MW, and PDQ-39) between the 2 groups before treatment (*P* > .05), and the 2 groups were balanced and comparable (Table 2).

**Table 2:** Comparison of pretreatment scale scores between 2 groups.

	rTMS(n = 40)	rTMS + rPMS(n = 40)	*P*
UPDRS-III	25 (21–35)	22 (17–26)	.057
10MW (m/s)	0.77 (0.68–0.96)	0.79 (0.73–0.96)	.509
TUG (s)	11.06 (9.62–12.51)	11.31 (10.15–13.36)	.697
PDQ-39	63 (36–80)	70 (42–90)	.180

10MW = 10-m walk timed test; PDQ-39 = Parkinson’s disease patient quality of life questionnaire; rPMS = repetitive peripheral magnetic stimulation; rTMS = repetitive transcranial magnetic stimulation; TUG = timed up-and-go test; UPDRS-III = Unified Parkinson’s Disease Rating Scale III.

### 3.2. Research results

#### 3.2.1. Efficacy comparison between the rTMS and rTMS + rPMS groups

Motor function (UPDRS-III, TUG, and 10MW) and quality of life (PDQ-39) improved after treatment in both treatment groups compared to before treatment (*P* < .05) (Table 3A). Comparison of the efficacy between the 2 groups showed better improvements in overall motor function (UPDRS-III) and quality of life (PDQ-39) scores in the rTMS + rPMS group than in the rTMS group. The difference in the therapeutic effect between the 2 groups was statistically significant(*P* < .05) (Table 3B).

**Table 3A:** Therapeutic efficacy of rTMS group and rTMS + rPMS group.

	rTMS group(n = 40)			rTMS + rPMS group(n = 40)		
	Before treatment	After treatment	*Pa*	Before treatment	After treatment	*Pb*
UPDRS-III	25 (21–35)	18 (13–22)	<0.001	22 (17–26)	14 (11–18)	<0.001
10MW	1.055 (0.86–1.23)	0.77 (0.68–0.96)	<0.001	0.91 (0.78–1.15)	0.79 (0.73–0.96)	<0.001
TUG	11.06 (9.62–12.51)	9.35 (8.03–11.60)	<0.001	11.31 (10.15–13.36)	8.69 (7.62–11.09)	<0.001
PDQ-39	63 (36–80)	35 (20–46)	<0.001	70 (42–90)	49 (23–79)	<0.001

10MW = 10-m walk timed test; TUG = timed up-and-go test; PDQ-39 = Parkinson’s disease patient quality of life questionnaire; rPMS = repetitive peripheral magnetic stimulation; rTMS = repetitive transcranial magnetic stimulation; UPDRS-III = Unified Parkinson’s Disease Rating Scale III.

*Pa*: Comparison of efficacy before and after treatment in the rTMS group.

*Pb*: Comparison of efficacy before and after treatment in the rTMS + rPMS group.

**Table 3B:** Comparison of efficacy between the rTMS and rTMS + rPMS groups.

	rTMS group(n = 40)	rTMS + rPMS group(n = 40)	
	Difference before and after treatment	Difference before and after treatment	*Pc*
UPDRS-III	6 (4–9)	10 (4–13.75)	0.035
10MW	0.16 (0.1–0.19)	0.12 (0.05–0.20)	0.363
TUG	1.95 (1.09–2.81)	2.28 (1.08–4.06)	0.403
PDQ-39	6 (4.25–8)	14 (8.5–18)	<0.001

10MW = 10-m walk timed test; PDQ-39 = Parkinson’s disease patient quality of life questionnaire; rPMS = repetitive peripheral magnetic stimulation; rTMS = repetitive transcranial magnetic stimulation; TUG = timed up-and-go test; UPDRS-III = Unified Parkinson’s Disease Rating Scale III.

*Pc*: Comparison of efficacy between rTMS and rTMS + rPMS groups before and after treatment.

#### 3.2.2. Exploration of the most suitable population for two treatment methods

##### 3.2.2.1. Efficacy comparison between early-stage and mid-late-stage subgroups

In the rTMS and rTMS + rPMS groups, motor function (UPDRS-III, TUG, and 10MW), and quality of life (PDQ-39) in the early and middle-late-stage subgroups improved before and after treatment; however, the difference between the early and middle-late-stage subgroups in both treatment groups was not statistically significant (*P* > .05) (Table 4).

**Table 4:** Comparison of efficacy between the early-stage and middle-late-stage subgroups.

	rTMS group		*Pa*	rTMS + rPMS group		*Pb*
	Early-stage subgroup(n = 16)	Middle-late-stage subgroup (n = 24)		Early-stage subgroup(n = 15)	Middle-late-stage subgroup (n = 25)	
	Difference before and after treatment	Difference before and after treatment		Difference before and after treatment	Difference before and after treatment	
UPDRS-III	7 (4–9)	5 (4–10)	0.817	6.5 (5.0–9.0)	5 (3–10.5)	0.764
TUG	0.14 (0.10–0.22)	0.14 (0.08–0.17)	0.699	0.12 (0.04–0.17)	0.11 (0.08–0.21)	0.862
10MW	1.64 (0.98–2.00)	1.98 (1.14–2.92)	0.081	2.20 (0.94–3.45)	4 (1.07–4.84)	0.089
PDQ-39	8 (5–12)	8 (7–10)	0.699	14 (11–18)	14.5 (9.0–26.5)	0.871

10MW = 10-m walk timed test; PDQ-39 = Parkinson’s disease patient quality of life questionnaire; rPMS = repetitive peripheral magnetic stimulation; rTMS = repetitive transcranial magnetic stimulation; TUG = timed up-and-go test; UPDRS-III = Unified Parkinson’s Disease Rating Scale III.

*Pa*: Comparison of efficacy between the early and middle-late-stage subgroups of the rTMS group.

*Pb:* Comparison of efficacy between the early and middle-late-stage subgroups of the rTMS + rPMS group.

##### 3.2.2.2. Efficacy comparison between male and female subgroups

In the rTMS and rTMS + rPMS groups, motor function (UPDRS-III, TUG, and 10MW), and quality of life of patients (PDQ-39) in the male and female subgroups improved before and after treatment; however, the differences between the male and female subgroups in both treatment groups were not statistically significant(*P* > .05) (Table 5).

**Table 5:** Comparison of efficacy between the male and female subgroups.

	rTMS group		*Pa*	rTMS + rPMS group		*Pb*
	Male subgroup(n = 18)	Female subgroup(n = 22)		Male subgroup(n = 25)	Female subgroup(n = 15)	
	Difference before and after treatment	Difference before and after treatment		Difference before and after treatment	Difference before and after treatment	
UPDRS-III	7 (5–12)	5 (4–8)	0.175	7 (5–9)	5 (4–7)	0.092
TUG	0.14 (0.10–0.19)	0.15 (0.08–0.19)	0.230	0.10 (0.06–0.16)	0.15 (0.15–0.21)	0.059
10MW	1.78 (1.18–2.05)	1.52 (0.98–2.90)	0.941	2.85 (1.09–4.04)	2.11 (0.94–2.60)	0.285
PDQ-39	8 (5–10)	8 (6–12)	0.080	14 (10–18)	14 (11–18)	0.774

10MW = 10-m walk timed test; PDQ-39 = Parkinson’s disease patient quality of life questionnaire; rPMS = repetitive peripheral magnetic stimulation; rTMS = repetitive transcranial magnetic stimulation; TUG = timed up-and-go test; UPDRS-III = Unified Parkinson’s Disease Rating Scale III.

*Pa*: Comparison of efficacy between the male and female subgroups of rTMS group.

*Pb*: Comparison of efficacy between the male and female subgroups of combination group.

##### 3.2.2.3. Efficacy comparison between the ≤60-year and >60-year age subgroups

In the rTMS and rTMS + rPMS groups, motor function (UPDRS-III, TUG, and 10 MW), and quality of life (PDQ-39) improved before and after treatment in patients with PD aged ≤60 years and >60 years. However, there was no statistically significant difference in efficacy between the treatment age subgroups of ≤60 years and >60 years in both treatment groups (*P* > .05) (Table 6).

**Table 6:** Comparison of efficacy between ≤60 years old and >60 years old subgroups

	rTMS group		*Pa*	rTMS + rPMS group		*Pb*
	≤60 years subgroup(n = 14)	>60 years subgroup(n = 26)		≤60 years subgroup(n = 12)	>60 years subgroup(n = 28)	
	Difference before and after treatment	Difference before and after treatment		Difference before and after treatment	Difference before and after treatment	
UPDRS-III	7.5 (6.5–12)	5 (4–9)	0.305	6 (4–9)	6 (4.5–9.5)	0.394
TUG	0.17 (0.11–0.21)	0.14 (0.08–0.18)	0.384	0.08 (0.04–0.15)	0.13 (0.10–0.21)	0.599
10MW	0.85 (1.04–2.33)	1.26 (1.06–2.46)	0.521	1.82 (0.50–4.11)	2.59 (0.11–4.00)	0.302
PDQ-39	7.0 (4.5–11.5)	8 (6–12)	0.384	12 (7–17)	15 (13–19)	0.066

10MW = 10-m walk timed test; PDQ-39 = Parkinson’s disease patient quality of life questionnaire; rPMS = repetitive peripheral magnetic stimulation; rTMS = repetitive transcranial magnetic stimulation; TUG = timed up-and-go test; UPDRS-III = Unified Parkinson’s Disease Rating Scale III.

*Pa*: Comparison of efficacy between ≤60 and >60 years of age in the rTMS group.

*Pb*: Comparison of efficacy between ≤60 and >60 years of age in rTMS + rPMS group

##### 3.2.2.4. Efficacy comparison between rigidity-predominant and tremor-predominant subtypes

A comparison of the effects of the two treatments in patients with rigidity-predominant and tremor-predominant PD showed improved motor function (UPDRS-III, TUG, and 10 MW) and quality of life (PDQ-39) before and after treatment in patients with rigidity-predominant and rigidity-predominant types of rTMS and rTMS + rPMS. Compared with patients with tremor-predominant PD, the UPDRS-III score of patients with rigidity-predominant PD improved more significantly, with a significant difference between the 2 subgroups (*P* < .05) (Table 7).

**Table 7:** Comparison of effects between rigidity-predominant and tremor-predominant types subgroups.

	rTMS group			rTMS + rPMS group		*Pb*
	Rigidity-predominant subtype (n = 18)	Tremor-predominant subtype (n = 12)		Rigidity-predominant subtype (n = 20)	tremor-predominant subtype (n = 11)	
	Difference before and after treatment	Difference before and after treatment	*Pa*	Difference before and after treatment	Difference before and after treatment	
UPDRS-III	8 (5–13)	4.0 (4.0–7.0)	0.037	7 (5–10)	5 (4–6)	0.030
TUG	0.18 (0.12–0.24)	0.09 (0.04–0.14)	0.180	0.13 (0.03–0.19)	0.10 (0.07–0.13)	0.711
10MW	1.88 (1.22–2.92)	1.46 (0.98–2.05)	0.149	2.86 (1.76–3.95)	1.53 (0.80–4.00)	0.261
PDQ-39	9 (6–13)	6.0 (5.0–9.0)	0.404	14 (12–19)	10 (7–10)	0.058

10MW = 10-m walk timed test; PDQ-39 = Parkinson’s disease patient quality of life questionnaire; rPMS = repetitive peripheral magnetic stimulation; rTMS = repetitive transcranial magnetic stimulation; TUG = timed up-and-go test; UPDRS-III = Unified Parkinson’s Disease Rating Scale III.

*Pa*: Comparison of efficacy between the rigidity-predominant and tremor-predominant subgroups of the rTMS group.

*Pb*: Comparison of efficacy between rigidity-predominant and tremor-predominant subgroups in the rTMS + rPMS group.

**Figure 2.**
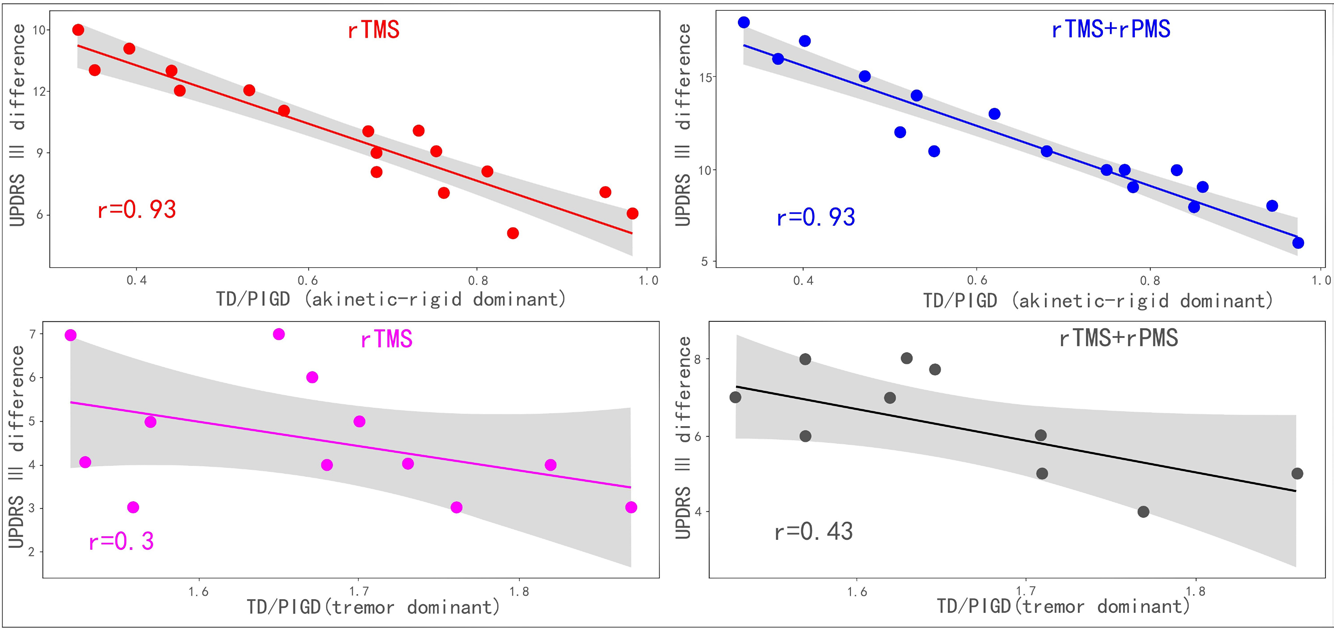
Correlations between the differences before and after UPDRS-III treatment and TD/PIGD ratios. The results showed that the UPDRS-III difference was negatively correlated with the TD/PIGD ratio. A comparison of the correlation coefficients revealed a higher correlation coefficient for the rigidity-predominant type than the tremor-predominant type in the rTMS and rTMS + rPMS groups. Therefore, the treatment effect was significantly greater for the rigidity-predominant type than the tremor-predominant type. PIGD = akinetic-rigid dominant, rPMS = repetitive peripheral magnetic stimulation, rTMS = repetitive transcranial magnetic stimulation, TD = tremor dominant, UPDRS-III = Unified Parkinson’s Disease Rating Scale III.

Correlation analysis was performed to investigate the difference between the UPDRS-III scores before and after treatment and the TD/PIGD ratio (Fig. 2). The difference before and after the UPDRS-III treatment was used as the dependent variable, and the TD/PIGD ratio was used as the independent variable. The results showed that the difference before and after the UPDRS-III treatment was negatively correlated with the TD/PIGD ratio. A comparison of the correlation coefficients revealed a higher correlation coefficient for the rigidity-predominant type than the tremor-predominant type in the rTMS group. In the rTMS + rPMS group, the correlation coefficient for the rigidity-predominant type was higher than that for the tremor-predominant type. Therefore, the treatment effect was significantly greater for the rigidity-predominant type than the tremor-predominant type.

### 3.3. Adverse reactions

The adverse reactions that occurred during this study included 3 cases of mild headaches and 1 case of neck pain. The adverse reactions were mild, short-lived, relieved after a short rest, and did not recur during retreatment. No serious adverse reactions occurred during the study, and no patient withdrew from the study because of adverse reactions.

## 4. Discussion

The motor symptoms of PD run through the entire disease process, and as the disease progresses, the motor symptoms gradually worsen, affecting the quality of life of PD patients. Currently, there are various ways to treat motor symptoms of PD in clinical practice,^[17]^ with the main approach being alternative therapies based on dopaminergic drugs.^[18]^ As the disease progresses, the efficacy of drugs gradually weakens and adverse reactions become more prominent. Most patients experience complications after 5 years of treatment,^[19]^ and even accelerate neurodegeneration. Therefore, there is an urgent need for new treatment plans to assist in the treatment of PD. Magnetic stimulation technology has the advantages of noninvasive, safe, easy to operate, and has good compliance, making it easy for patients to accept and use in the clinical treatment of PD.

### 4.1. Exploration of magnetic stimulation mechanism

rTMS is an important non pharmacological treatment for PD.^[20]^ The regulation of PD by rTMS is the result of the interaction of multiple but not mutually exclusive mechanisms, mainly including: (1) regulating cortical excitability: Studies have shown that high-frequency rTMS can increase the excitability and activity of the cortex in areas with decreased cortical function in patients with PD. By applying the cortical basal nucleus thalamic cortical circuit to regulate function, cortical excitability can approach a normal state and improve clinical symptoms in patients with PD.^[21]^ (2) Regulating the plasticity response of neural networks: rTMS can enhance interactions between cortical regions.^[22,23]^ (3) Activating the dopamine pathway in the midbrain striatum promotes dopamine release.^[24,25]^ (4) Promotion of brain-derived neurotrophic factor synthesis.^[26]^

rPMS refers to a magnetic stimulation mode that uses magnetic stimulation coils to directly act on peripheral nerves and muscles, to promote the recovery of motor function. At present, it has been applied to various clinical diseases such as stroke, dysphagia, spinal cord injury, neurogenic urinary system diseases, pain, etc.^[27–30]^ Research has shown that rPMS also plays a role in the motor symptoms and hunchback in PD.^[31,32]^The principle of rPMS in treating motor disorders is not fully understood, and current research suggests the following aspects: (1) The pulse neural signals generated by rPMS are transmitted from bottom to top, activating and reshaping the spinal cord and brain of patients, thereby improving their sensory and motor functions in patients with neurological injuries^[33]^; (2) The large number of ascending sensory signals triggered by rPMS can increase the subcortical level sensory input, thereby widely activating brain regions and the cerebellum, such as the prefrontal cortex, premotor cortex auxiliary motor area, cingulate cortex, and parietal cortex in patients,^[34]^ increasing the amplitude of motor evoked potentials and enhancing the activation of the sensory cortex.^[35]^ (3) Enhance the excitability of peripheral nerves^[36]^; (4) Induce peripheral muscle contraction and affect local blood flow, thereby affecting ion content in the blood.^[37]^ Research has found that the activity of the distal lower limb muscles in patients with PD is significantly reduced,^[38]^ especially affecting the tibialis anterior muscle, which is innervated by the common peroneal nerve. Electrical stimulation of the common peroneal nerve can reduce falls, improve walking speed and efficiency, improve gait safety, and improve quality of life.^[39]^ The common peroneal nerve is located on the posterior surface of the fibular head, which is easier to stimulate than the tibial and medial calcaneal nerves. Therefore, we chose to stimulate the patient’s common peroneal nerve. Research has shown that rPMS with a frequency of 1Hz can inhibit cortical excitability, whereas rPMS with a frequency of 25 Hz can increase cortical motor excitability.^[40]^ This is consistent with the mechanism of rTMS in the treatment of the motor symptoms of PD. Therefore, in this study, we chose to stimulate the common peroneal nerve with rPMS at 25 Hz to treat the motor symptoms of Parkinson’s.

The combination of rTMS and rPMS is achieved through the action of TMS on the central nervous system, regulating cortical excitability, activating corresponding brain regions, and improving central nervous system function remodeling. PMS activates the motor cortex through the sensory conduction system from bottom to top, promoting targeted learning and cognition, and effectively activating the motor cortex to generate positive feedback information input to the central nervous system, thereby promoting the remodeling of brain neural function. PMS can directly increase the excitability of stimulating nerves. The combination of the 2 forms a circuit,^[41]^ that can achieve excitation and activation of the entire sensory motor circuit, enhance brain plasticity and neural pathway remodeling, and improve rehabilitation efficiency. Animal experiments have shown that combination therapy can upregulate the expression of brain plasticity related proteins, increase regional brain activity, and promote neurological function recovery.^[42]^

### 4.2. Observation of therapeutic effects

#### 4.2.1. Observation on the therapeutic effect of rTMS

In this study, we used high-frequency (10 Hz) rTMS stimulation of the M1 region. The meta-analysis results showed that high-frequency stimulation of the M1 region can reduce the UPDRS-III score in patients and regulate the expression of dopamine, interferon, brain-derived neurotrophic factors, etc. High frequency 10 Hz stimulation of the M1 region has a more effective effect on Parkinson’s movement.^[43–45]^ Consistent with the findings of this study, high-frequency rTMS stimulation of the M1 region significantly improved overall motor function (UPDRS III), balance function (TUG), movement speed (10MW), and quality of life (PDQ-39) in Parkinson’s.

#### 4.2.2. Observation on the therapeutic effect of rTMS + rPMS

The scheme used in this study was a novel magnetic stimulation scheme. Although there have been no reports of rTMS + rPMS treatment in PD, there have been many studies on the rehabilitation treatment of stroke.

The results of this study showed that the motor symptom score (UPDRS III, 10MW, TUG) and quality of life score (PDQ-39) improved in the rTMS + rPMS group after treatment, with statistical differences between each indicator (*P* < .05). Compared with the rTMS group, the rTMS + rPMS group showed more improvements in the overall motor performance score (UPDRS III) and quality of life score (PDQ-39) (*P* < .05). This result indicates that rTMS + rPMS has a synergistic effect on the overall improvement of motor symptoms in Parkinson’s, which is consistent with reports on other diseases.

### 4.3. Exploration of the optimal population

Previous studies have demonstrated that different characteristics of patients with PD can affect treatment efficacy.^[46–48]^ Therefore, the present study conducted a categorical analysis of the data from the rTMS and combined magnetic stimulation treatment groups. According to different clinical manifestations (mainly rigidity and tremor), disease severity (early and middle-late), sex (male and female), and treatment age (≤60 years and >60 years), the treatment effect was checked to determine the most suitable population for the two treatment methods. In the present study, in terms of overall motor function (UPDRS-III), the efficacy of rTMS and rTMS + rPMS differed between patients with rigidity-predominant PD and tremor-predominant PD, with more obvious improvement in the rigidity-predominant type (*P* < .05); however, the therapeutic effect did not differ among PD patients with different disease courses, sexes, and treatment ages (*P* > .05). Therefore, the different disease stages, sex, and treatment age did not influence the efficacy of the two treatment methods. This can be in a sense that the 2 therapies work in all age groups, all gender and irrespective of the disease stage with varying levodopa equivalent daily doses as well. The subgroups showed no significant differences in specific motor signs (10 MW, TUG), such as exercise speed (10MW, TUG) or quality of life (PDQ-39) (*P* > .05). However, this result cannot be ruled out because of the small sample size of our study. According to the above data analysis, patients with rigidity-predominant PD as the main type were the most suitable group for the two treatment methods.

### 4.4. The innovation and limitations of this study

This study administered 2 weeks of magnetic stimulation treatment, considering the patient’s time, economy, safety, and compliance, as well as the treatment efficacy and the fact that the treatment could be repeated. These findings provide a new treatment strategy and a basis for the treatment of PD. We will further increase the sample size and combine functional magnetic resonance imaging and electrophysiological examinations to study the impact of this treatment plan on PD and provide a reference for its clinical application. Future research should also focus on establishing a standardized system for magnetic stimulation treatment and tailoring individualized treatment plans for PD patients.

This study had certain limitations to this study: (1) Due to the small sample size, no subgroup effect comparison study was conducted between the treatment effects of rTMS and rTMS + rPMS; (2) Studies show that the efficacy of rTMS in treating PD can be maintained for 1 month, which may be related to the follow-up time.^[49,50]^ Owing to time constraints, the patient was not further tracked and followed up. (3) Due to the difficulty in collecting data from PD patients with restricted activity in OFF state, all assessments were completed in ON phase. For future research, we will increase research on the efficacy of rTMS in OFF state and further explore the efficacy of rTMS. Future research should include a larger sample size, and further tracking and evaluation of the long-term effects of treatment. Future research should combine multimodal neuroimaging and electrophysiological examinations to further explore the potential mechanisms of rTMS combined with rPMS in the treatment of PD.

## 5. Conclusion

Both HF-rTMS and rTMS + rPMS improved movement symptoms and quality of life in patients with PD. rTMS + rPMS was more effective than rTMS alone in improving overall motor function (UPDRS III) and quality of life (PDQ-39).

The type of motor symptoms may be a factor influencing the efficacy of the two treatment methods, and PD patients with rigidity-based type as the main type may be the most suitable group for these two treatment methods.

The therapies work in all age groups, all gender and irrespective of the disease stage with varying levodopa equivalent daily doses as well.

## Acknowledgments

We thank Yajun Wu for assistance with the clinical study and the National Natural Science Foundation of China and Science and Technology Project of the Jiangsu Provincial Health Commission. We would like to thank Editage (www.editage.cn) for English language editing.

## Author contributions

**Data curation:** Junrui Li.

**Formal analysis:** Tian Xu.

**Methodology:** Haiqing Shen.

**Project administration:** Yongcheng Jiang, Xinjue Wang, Lihua Shen.

**Writing – original draft:** Peili Sun.

**Writing – review & editing:** Xiaosu Gu.
